# Genome Sequence of *Escherichia coli* Tailed Phage Utah

**DOI:** 10.1128/genomeA.01494-16

**Published:** 2017-03-30

**Authors:** Justin C. Leavitt, Alexandra J. Heitkamp, Ananda S. Bhattacharjee, Eddie B. Gilcrease, Sherwood R. Casjens

**Affiliations:** a Biology Department, University of Utah, Salt Lake City, Utah, USA; b Pathology Department, Division of Microbiology and Immunology, University of Utah School of Medicine, Salt Lake City, Utah, USA; c Department of Civil and Environmental Engineering, University of Utah, Salt Lake City, Utah, USA

## Abstract

*Escherichia coli* bacteriophage Utah is a member of the chi-like tailed phage cluster in the *Siphoviridae* family. We report here the complete 59,024-bp sequence of the genome of phage Utah.

## GENOME ANNOUNCEMENT

The lytic double-stranded DNA (dsDNA) tailed bacteriophage Utah was isolated in 2015 at the University of Utah as a laboratory contaminant that makes clear plaques on a lawn of *Escherichia coli* SKB178 (1). It also makes smaller plaques on *Salmonella enterica* serovar Typhimurium strain LT2 and infects only flagellated *Salmonella* cells (data not shown). Its virion morphology was determined by negative-staining transmission electron microscopy, which revealed an isometric head that is hexagonal in outline and about 60 nm in diameter, a 230-nm-long flexible noncontractile tail that has about 45 transverse striations, and a single long curly tail fiber.

Phage Utah was propagated on *E. coli* SKB178 (1), and its DNA was sequenced by Illumina MiSeq 150-bp paired-end run methodology with a 350-bp insert library at the High Throughput Genomics Core Facility, University of Utah. Quality-controlled trimmed reads were assembled to a single linear sequence contig with 20-fold coverage using Geneious 9.0.5 (2). Dideoxynucleotide sequencing runs (3) primed to run off the ends of the virion DNA molecule showed that the linear assembled sequence represents the complete phage Utah genome and that its chromosome has 12-bp 5′-overhanging cohesive ends, with the sequence 5′-GGTGCGCAGAGC at the left (5′) end. The 59,024-bp-long phage Utah genome has 56.4% G+C. We annotate 74 genes in the genome, which include large terminase, portal, prohead protease, major capsid, and tail tape measure virion assembly genes, as well as lysis genes and DNA metabolism genes that encode a putative helicase, primase, and DNA polymerase.

The genome sequence shows that phage Utah is a close relative of phage chi (χ) and belongs to the chi-like phage cluster (4); it is 90.4% identical to chi in nucleotide sequence by the DNA Strider alignment algorithm (5). Its closest known relative is *Salmonella* phage iEPS5 at 96.2% overall identity. The completely and nearly completely sequenced members of this phage cluster currently include phage Utah, *Salmonella* phages chi (accession no. KM458633) (6), iEPS5 (accession no. KC677662) (7), SPN19 (accession no. JN871591), SPN35 (accession no. KR296689), SPN37 (accession no. KR296691), FSL_SP-019, FSL_SP-30, FSL_SP-039, FSL_SP-088, FSL_SP-099, and FSL_SP-124 (accession no. KC139571 to KC139631, KC139519, KC139514, KC139512, KC139667 to KC139680, and KC139515, respectively) (8), BP21C (accession no. AIT13784), 118970_sal1 (accession no. KU927500), and a phage that apparently contaminated the *S. enterica* DT104 genome sequencing project (accession no. CVKM01000024, genome project PRJEB2189), as well as *Enterobacter cancerogenus* phage Enc34 (accession no. JQ340774) (9), *Providencia stuartii* phage RedJac (accession no. JX296113) (10), and *Proteus mirabilis* phage pPM_01 (accession no. KP063118). The phages in this group that have been examined (chi, iEPS5, and Utah) adsorb specifically to flagella and use active flagella as their receptor for adsorption (7, 11–13). These chi-like phages have similar gene contents, gene orders, and genome sizes (between 58 and 61 kbp), and they form a very well-defined cluster that is only distantly related to other described phage types (4, 6, 8). Interestingly, among the chi-like phage group’s closest, but still quite distant, relatives are the *Xylella fastidiosa* phages Salvo and Sano (14), and these *xylella* phages utilize a different external cell structure, type IV pili, as receptors.

### Accession number(s).

The complete genome sequence of phage Utah is available in the GenBank database with accession number KY014601.
